# Lipid profiling of RON and DEK-dependent signaling in breast cancer guides discovery of gene networks predictive of poor outcomes

**DOI:** 10.3389/fonc.2024.1382986

**Published:** 2024-09-16

**Authors:** Sara Vicente-Muñoz, James C. Davis, Adam Lane, Andrew N. Lane, Susan E. Waltz, Susanne I. Wells

**Affiliations:** ^1^ Translational Metabolomics Facility, Division of Pathology and Laboratory Medicine, Cincinnati Children’s Hospital Medical Center, Cincinnati, OH, United States; ^2^ Department of Pediatrics, University of Cincinnati, Cincinnati, OH, United States; ^3^ Department of Cancer Biology, College of Medicine, University of Cincinnati, Cincinnati, OH, United States; ^4^ Division of Bone Marrow Transplantation and Immune Deficiency, Cincinnati Children’s Hospital Medical Center, Cincinnati, OH, United States; ^5^ Department of Toxicology and Cancer Biology, University of Kentucky, Lexington, KY, United States; ^6^ Research Service, Cincinnati Veterans Affairs Medical Center, Cincinnati, OH, United States; ^7^ Division of Oncology, Cincinnati Children’s Hospital Medical Center, Cincinnati, OH, United States

**Keywords:** breast cancer, recurrence, metastasis, RON, DEK, NMR spectroscopy, lipidomics

## Abstract

Recurrent and metastatic breast cancer is frequently treatment resistant. A wealth of evidence suggests that reprogrammed lipid metabolism supports cancer recurrence. Overexpression of the RON and DEK oncoproteins in breast cancer is associated with poor outcome. Both proteins promote cancer metastasis in laboratory models, but their influence on lipid metabolite levels remain unknown. To measure RON- and DEK-dependent steady-state lipid metabolite levels, a nuclear magnetic resonance (NMR)-based approach was utilized. The observed differences identified a lipid metabolism-related gene expression signature that is prognostic of overall survival (OS), distant metastasis-free survival (DMFS), post-progression survival (PPS), and recurrence-free survival (RFS) in patients with breast cancer. RON loss led to decreased cholesterol and sphingomyelin levels, whereas DEK loss increased total fatty acid levels and decreased free glycerol levels. Lipid-related genes were then queried to define a signature that predicts poor outcomes for patients with breast cancer patients. Taken together, RON and DEK differentially regulate lipid metabolism in a manner that predicts and may promote breast cancer metastasis and recurrence.

## 1 Introduction

Advances in treatments have resulted in an overall 90% 5-year survival rate for breast cancer patients (1). However, a large gap remains between survival rates of those with localized versus distant metastatic breast cancer. While localized breast cancer has a 98% survival rate, distant metastatic breast cancer has a 31% survival rate. In addition, the 10-year survival rate for breast cancer decreases from 85% to 46.9% for local recurrence and to less than 5% for distant recurrence (2–4). This highlights an unmet need to identify markers and drivers of metastatic and recurrent breast cancer to improve currently unacceptable outcomes.

Herein, we focus on two genes that have already been associated with poor outcomes in patients with breast cancer. RON is a receptor tyrosine kinase overexpressed in over 50% of breast cancers independent of subtype (5), and stimulates cancer, metastasis, and recurrence (6). DEK is a chromatin-associated oncogene that also promotes breast cancer stemness and metastasis and can be stimulated by RON signaling (7). Together, high DEK and RON expression levels were strongly linked to poor outcomes in human subjects (5, 8). Previous studies have shown that fatty acids can regulate tumor cell growth, invasion, and progression both positively and negatively (9–12). In addition, increases in cholesterol biosynthesis and cholesterol levels have been associated with RON-driven breast cancer stem cell (BCSC) phenotypes (6, 13). Lipid metabolism is the new frontier of cancer biomarkers and targets (14) and might be a potential driver of RON- and DEK-associated metastasis and recurrence. We hypothesized that RON and DEK upregulation in breast cancer is required for lipid metabolism that is linked to poor outcomes.

We have used ^1^H-NMR (nuclear magnetic resonance) spectroscopy to determine relative abundances of different intact lipids in families defined by (i) headgroup (phosphatidylcholine (PC), phosphatidylethanolamine (PE)) and average degree of fatty acyl chain unsaturation, (ii) sphingomyelin, and (iii) cholesterol, rather than individual lipid species. This provides a broad overview of the complex lipids detectable in cells (15, 16). We found that breast cancer cells with RON and DEK loss of function harbor independent dysregulation of defined lipid metabolites, including cholesterol, unsaturated fatty acids, glycerol, sphingomyelin, and glycerophospholipids. By examining relevant transcriptionally regulated enzymes, we identified a lipid metabolism-related gene signature that predicts breast cancer recurrence, and also poor outcomes for lung, ovarian, and gastric cancer. These data also support a role for RON and DEK in energy metabolism to support breast cancer recurrence and metastasis.

## 2 Materials and methods

### 2.1 Cell culture

The well-characterized and widely published R7 (control), R7sgRON (RON targeted), and R7shDEK (DEK targeted) murine breast cancer cell lines were cultured as previously described (7, 17) in complete Dulbecco’s Modified Eagle’s Medium (DMEM) containing 5% FBS, 1% penicillin–streptomycin, and 0.2% fungizone. R7sgRON cells were obtained from serial dilution of px458-sgRON-transfected R7 cells. Px458 was obtained from Addgene (48138), whereas RON sgRNA was obtained from IDT (sgRON sequence: ACCTGCAGCTCACCCTTCTAC). R7shDEK cells were maintained with 1 μg/mL of puromycin for selection. For NMR experiments, cells were seeded in 10-cm plates in complete DMEM containing 5% dialyzed FBS.

### 2.2 Cell collection and processing

The three isogenic breast cancer cell lines were plated and incubated for 24 h. At the time of collection, cells were 80%–90% confluent; the medium was aspirated, and the cells were washed and quenched as per prior protocols (18). Polar and non-polar metabolites were extracted using the solvent partition method with acetonitrile: water: chloroform (CH_3_CN: H_2_O: CHCl_3_) at ratios 2: 1.5: 1 (V/V) (18, 19). A mixture of chloroform and methanol (1:1) containing 1 mM butylated hydroxytoluene (BHT) was added to the lipid fraction for storage at −80°C. The upper aqueous phases (polar metabolites) were lyophilized (CentriVap Labconco), and the lower organic phases (lipidic non-polar metabolites) were dried in a SpeedVac at room temperature.

### 2.3 NMR spectroscopy

Dried organic phases (lipids) were reconstituted in 220 µL of 100% methanol-d4 containing 0.05% v/v of tetramethylsilane (TMS) (Cambridge Isotopes Lab, Andover, MA), vortexed, and centrifuged at room temperature. 200 µL of the supernatant was transferred into a 3-mm NMR tube. All NMR spectra were recorded at 288 K on a Bruker Avance III HD 600-MHz spectrometer (Bruker BioSpin) equipped with a 5-mm Broad Band Observed (BBO) Prodigy probe. For each sample, one-dimensional (1D) ^1^H-NMR experiments were acquired using the noesygppr1d pulse sequence with presaturation of the residual water resonance using a 25-Hz bandwidth, 512 transients, a 15-ppm spectral width, a 4.0-s relaxation delay, and a 2.0-s acquisition time resulting in 44,640 data points. Prior to Fourier transformation, each ^1^H spectrum was zero-filled to 128 K data points and apodized with a 1-Hz exponential line-broadening function. All spectra were recorded and transformed with the use of TopSpin 3.6.2 software (Bruker BioSpin, USA) and processed (phased and baseline corrected) using MestReNova software (MNova v12.0.3, Spain). Spectra were internally calibrated to the methyl group of the TMS at 0 ppm. Representative lipid families (glycerophospholipids, sterols, sphingolipids, glycerophospholipids, and fatty acids) were identified and assigned by using in-house databases, pure standards, and literature reports (16, 20, 21). Additionally, for selecting samples, 2D ^1^H-^1^H TOtal Correlation SpectroscopY (TOCSY) experiments were recorded to facilitate and confirm the identification of analytes. The area of each assigned lipid class was manually integrated using the global spectra deconvolution (GSD) algorithm available in MestReNova software (MNova v12.0.3, Spain), as previously described (18).

### 2.4 Statistical analysis

To assess the relative abundance of each species of lipids identified compared with the R7 (control) group, each peak area was internally normalized to the area of the (CH_3_)_3_-N^+^ choline resonance at 3.22 ppm in the same spectrum. Principal component analysis (PCA) was carried out on the data scaled to unit variance by dividing each variable by its standard deviation (SD) using the R software. Univariate statistical analysis was used to determine the relevant spectral regions responsible for the discrimination between the groups. One-way ANOVA was used to generate pairwise comparisons between the R7, R7sgRON and R7shDEK groups. The Benjamini–Hochberg procedure (22) was used to control the false discovery rate of the pairwise comparison at q = 0.05. The control group was set to 1 for comparison, and the data were expressed as fold change of the relative amount of the different lipid adducts. Data are displayed as mean ± standard error of the mean (SEM).

### 2.5 Design and validation of a predictive gene signature

Select genes encoding enzymes involved in lipid metabolism (fatty acids, cholesterol, steroids, sphingolipids, phospholipids, triglycerides, glycolipids, and cardiolipins) were utilized to stratify breast cancer patient outcomes with respect to relapse-free survival (RFS) in the Gene Expression Omnibus-derived KMplot datasets (23). A comprehensive list of genes associated with these lipid-related pathways was obtained (24, 25). This initial list was then refined to generate a gene signature based on the log-rank p-value for RFS, using the KMplot webtool. Stratification into low and high gene expression groups was performed using a sliding cutoff approach from the KMplot webtool which optimizes hazard ratio (HR) values. Genes with worse outcomes from lower expression had their values inverted for the gene signature. Genes whose expression statistically significantly stratified RFS (p value < 0.05) were used to construct the lipid gene signature through the arithmetic mean of each gene. Each signature was subjected to a sliding cutoff. Additionally, the gene signature was used to test breast cancer patient overall survival (OS), distant metastasis-free survival (DMFS), and post-progression survival (PPS) in the Gene Expression Omnibus-derived KMplot datasets (23). The signature was then validated using The Cancer Genome Atlas (TCGA) Pan Cancer dataset (26) Overall Survival (OS) and Progression Free Survival (PFS). Finally, we tested the gene signatures for their capacity to predict response to chemotherapy in node-positive breast cancer patients using receiver-operator characteristic (ROC) analysis from the ROCplot webtool (27) analyzing GEO-derived breast cancer patient data. For the ROC analysis, genes that were upregulated and led to worse outcomes were tested separately from genes that were downregulated. Finally, the gene signature was tested using the KMplot dataset for ovarian and lung cancer (28, 29) and the ROCplot webtool for 6-month survival in ovarian cancer (27).

## 3 Results

### 3.1 Lipidomic profile of breast cancer by ^1^H-NMR spectroscopy

**Figure 1:**
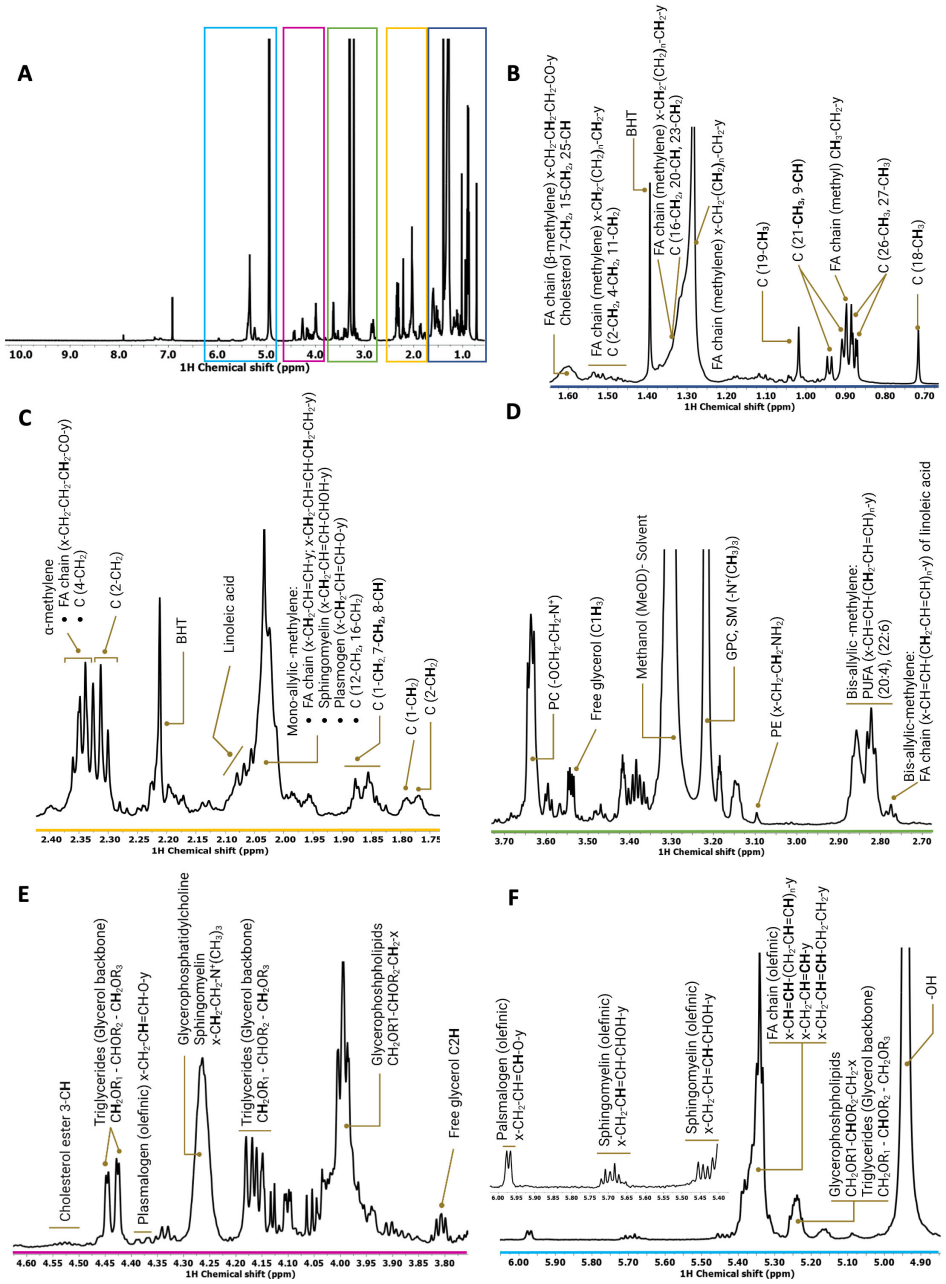
Representative ^1^H spectrum derived from the whole-cell lipid extract of R7sgRON cell line. **(A)** Full ^1^H spectrum acquired in a Bruker Avance spectrometer operating at 600 MHz. There were 512 transients that were acquired at 288 K, apodized using 1-Hz line broadening exponential, Fourier transformed, phased, and baseline corrected. **(B–F)** Expansion of different regions of the ^1^H spectrum (0.00 ppm–6.00 ppm) of the total lipid fraction showing the assignments of the lipid classes identified. C, cholesterol; FA, fatty acids; PC, phosphocholine; SM, sphingomyelin; BHT, butylated hydroxytoluene (solvent, antioxidant).

To qualitatively and quantitatively define RON- and DEK-dependent reprogramming of lipid metabolites, NMR spectroscopy was applied to R7 breast cancer cells and the corresponding RON or DEK knockdown cell lines. Specifically, proton (^1^H) NMR determined lipid composition in the presence and absence of RON and DEK. Despite the inherent width of individual lipid resonances, as well as the wide variety and complexity of the lipid species, good-quality spectra were obtained for all samples. A representative 1D ^1^H NMR spectrum of the total lipid fraction of R7sgRON is shown in **Figure 1A** where the most representative lipid families were identified and assigned. As previously described (16, 30, 31), the ^1^H-NMR resonances of the subunits of the different lipid classes were distributed as follows: the methyl (CH_3_) and methylene (CH_2_) resonances from cholesterol and the acyl chains are located from 0.65 ppm to 3.00 ppm (**Figures 1B–D**). The spectral region between 3.05 and 5.25 ppm (**Figures 1D–F**) comprises the phospholipid head group and glycerol backbone moieties of the glycerophospholipids. The vinyl proton peaks resound between 5.30 ppm and 6.00 ppm (**Figure 1F**). Therein, we determined the relative abundance of different subunits of complex lipids, including sterols (e.g., cholesterol), sphingolipids, glycerophospholipids, and fatty acids. We applied principal component analysis (PCA) as an exploratory tool to assess the separation of the three groups based on their lipid profiles (**Supplementary Figure S1**). Clear separation observed in the PCA plot provides visual evidence of distinct lipid patterns among the groups (**Supplementary Figure S1A**). The corresponding loading plot identified the most important variables driving the PCA model, correlations between variables, and species contributing to the group separation (**Supplementary Figure S1B**). Each individual lipid region was then evaluated statistically using one-way ANOVA.

### 3.2 RON promotes cholesterol biosynthesis

**Figure 2:**
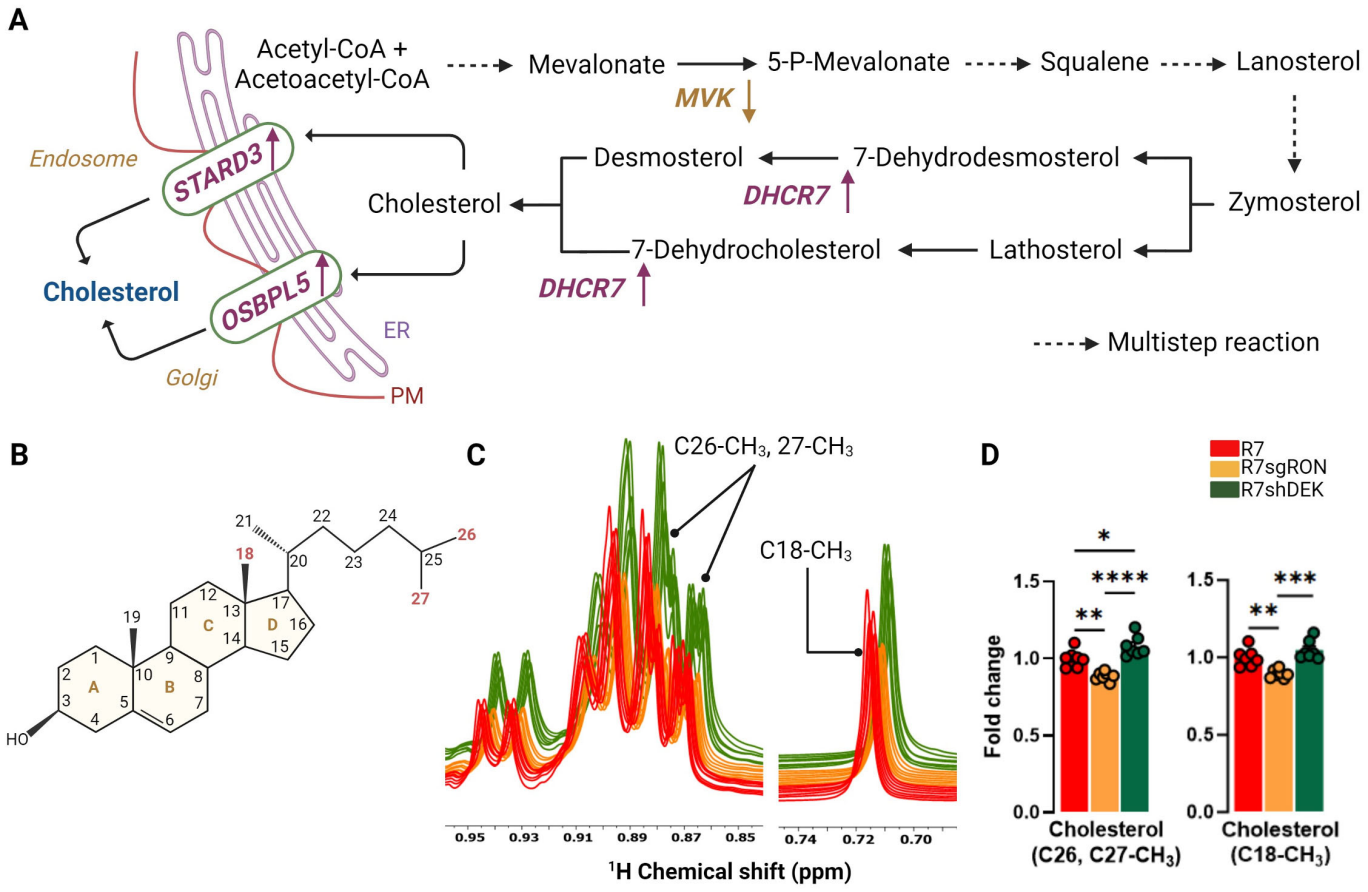
RON stimulates cholesterol metabolism. **(A)** Cholesterol biosynthesis pathway. Multistep reactions are illustrated with discontinuous arrows. Relevant enzymes from the KMplot.com-derived lipid metabolism gene signature are shown where appropriate, with an upward arrow indicating that gene induction is associated with worse patient outcomes and a downward arrow indicating that gene suppression is associated with worse patient outcomes. **(B)** Chemical structure of cholesterol. The carbon positions highlighted in red represent the cholesterol atoms quantified by NMR and compared between cell lines. **(C)** Comparison of 1D ^1^H-NOESY spectra of the lipid fraction from R7, R7sgRON, and R7shDEK corresponding with the cholesterol peaks analyzed by ^1^H-NMR. **(D)** Intracellular fold change of cholesterol measured from C26, C27-CH_3_, and C18-CH_3_ carbon positions and quantified by ^1^H-NMR and expressed as a fold change of the control group (R7). Bar plots indicate mean ± SEM. Replicates of each cell line are shown as individual dots (n = 7). Statistical significance was assessed using one-way ANOVA. *P ≤ 0.05; **P ≤ 0.01; ***P ≤ 0.001; ****P ≤ 0.0001. PM, plasma membrane; MVK, mevalonate kinase; DHCR7, 7-dehydrocholesterol reductase; STARD3, StAR-related lipid transfer domain containing 3; OSBPL5, oxysterol binding protein like 5.

Cholesterol biosynthesis is associated with increased breast cancer stem cell potential and increased recurrence (32) and entails a series of highly complex reactions that occur in the endoplasmic reticulum (ER) (33). The process begins with Acetyl-CoA and eventually results in a four-ring structure with a side chain and a total of 27 carbons (**Figures 2A, B**). Once formed, cholesterol is transported via non-vesicular and vesicular mechanisms (34). Vesicular transport of cholesterol occurs through organelle membranes, such as in endosomes and the Golgi apparatus, to the cellular membrane with the participation of sterol and oxysterol trafficking proteins (35, 36) (**Figure 2A**). Cholesterol upregulation is a poor prognostic factor in breast cancer and is associated with shortened relapse-free survival. Cholesterol also acts as an estrogen receptor agonist via its metabolite 27-hydroxycholesterol, supporting recurrence and metastasis progression (13, 37). Cholesterol resonances from ^1^H spectra were integrated, quantified, and used to determine cholesterol levels in the R7, R7sgRON, and R7shDEK cell lines. The methyl (CH_3_) cholesterol resonance at positions C18, and C26, C27, showed that RON loss decreased cholesterol levels. In contrast, DEK loss did not impact cholesterol levels and even elevated them compared with the R7 control (**Figures 2C, D**). In line with our previous study (6) wherein RON promoted key genes and enzymes in the cholesterol biosynthesis pathway as well as the incorporation of glucose-derived carbon into cholesterol, this establishes that RON (but not DEK) overexpression contributed to the promotion of cholesterol biosynthesis.

### 3.3 RON expression in breast cancer increases sphingomyelin levels

**Figure 3:**
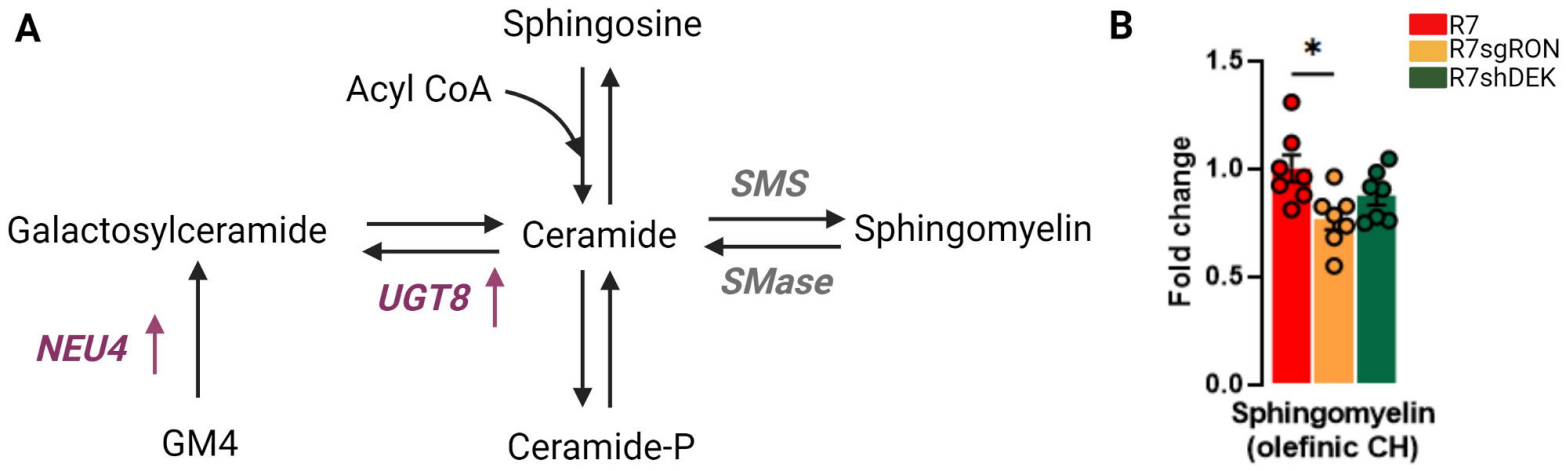
RON knockout decreases sphingomyelin levels. **(A)** Schematic representation of the synthesis of sphingomyelin and other derivatives of ceramide. Translation products from the KMplot.com-derived lipid metabolism gene signature have been added where appropriate, with an upward arrow indicating that upregulation of this gene is associated with worse patient outcomes. **(B)** Intracellular fold change of sphingomyelin measured from sphingomyelin olefinic CH ^1^H-NMR. Bar plots indicate mean ± SEM. Replicates of each cell line are shown as individual dots (n = 7). Statistical significance was assessed using one-way ANOVA. *P ≤ 0.05. GM4, N-acetylneuraminyl-galactosylceramide; NEU4, N-acetyl-alpha-neuraminidase 4; UGT8, UDP glycosyltransferase 8; SMS, sphingomyelin synthase.

Sphingolipids are important structural constituents of cell membranes. Ceramide is a precursor and essential intermediate in the synthesis and metabolism of all sphingolipids (**Figure 3A**). Sphingomyelin is produced by the transfer of phosphorylcholine from phosphatidylcholine to ceramide and constitutes the most abundant sphingolipid in mammalian cells. Sphingomyelin can also be hydrolyzed to produce ceramide, a pro-apoptotic molecule (**Figure 3A**). Quantification of the ^1^H-NMR peaks at 5.70 ppm corresponding to the olefinic CH of the sphingomyelin showed that the R7sgRON cells had decreased sphingomyelin levels compared with both the R7 and R7shDEK cell lines (**Figure 3B**). This implies that RON, but not DEK, potentially promotes sphingomyelin synthesis and breast cancer progression. Both ceramide and sphingomyelin act as regulatory molecules that inversely control cellular proliferation and tumor progression. Increased production (or reduced degradation) of sphingomyelin indirectly promotes cell proliferation due to decreased ceramide levels. Moreover, aberrant accumulation of sphingomyelin in the plasma membrane impairs membrane fluidity and permeability. As a consequence, sphingomyelin not only decreases cell–cell communication in favor of uncontrolled proliferation and invasion but also impairs anticancer drug influx, contributing to chemotherapeutic resistance (38).

In addition, several sphingolipid enzymes play a key role in the ceramide–sphingomyelin homeostasis and cancer progression. UDP glycosyltransferase 8 (UGT8) mediates the synthesis of galactosylceramide from ceramide using UDP-galactose as a donor (**Figure 3A**). Higher UGT8 mRNA and protein levels were observed in breast cancer metastases to the lung compared with their respective primary tumors (39). The overexpression of UGT8 and accumulation of its product galactosylceramide are associated with increased aggressiveness and worse prognosis in breast cancer (39, 40). Notably, UGT8 has already been identified as part of a six-gene signature that correlates with a higher risk for developing lung metastases and has been validated in three independent cohorts of breast cancer patients (41).

Finally, sphingomyelin synthase (SMS) mediates the formation of sphingomyelin from ceramide. High SMS expression leads to the concomitant accumulation of sphingomyelin and reduction of ceramide. An imbalance in ceramide–sphingomyelin homeostasis stimulates cancer cell proliferation by reducing ceramide-related apoptosis and induces tumor invasiveness and migration by enhancing epithelial-to-mesenchymal transition (EMT) (42, 43).

### 3.4 DEK regulates glycerophospholipid levels and free glycerol availability

**Figure 4:**
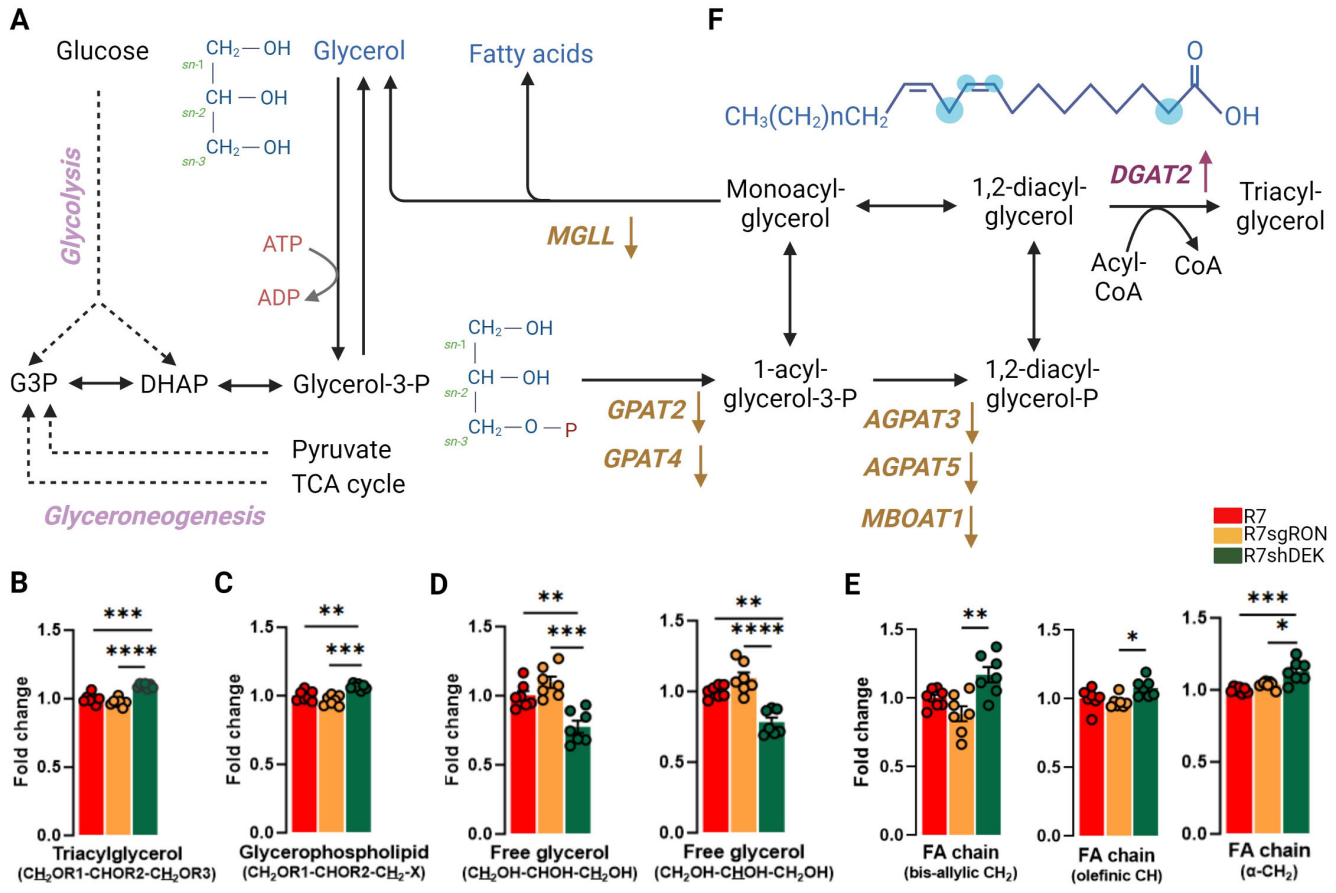
DEK knockdown increases glycerophospholipids and FA while decreasing free glycerol. **(A)** Schematic representation of metabolic processes involved in the production of glycerol, glycerophospholipids, and fatty acids. Translation products from the KMplot.com-derived lipid metabolism gene signature have been added where appropriate, with an upward arrow indicating that upregulation of this gene is associated with worse patient outcomes and a downward arrow indicating that downregulation of this gene is associated with better patient outcomes. **(B)** Intracellular fold change of the protons located at positions C1 and C3 of the glycerol backbone (CH_2_OR1–CHOR2–CH_2_OR3) of the TAG. **(C)** The glyceryl C3H_2_ group (CH_2_OR1–CHOR2–CH_2_-X) of glycerophospholipids. **(D)** The NMR peaks derived from free glycerol, both C1H_3_ and C3H_3_ and C2H_2_ resonances. **(E)** Analysis of the bis-allylic (–CH_2_) groups, which is specific for linoleic acid, the olefinic (–CH), and α-methylene fatty acid group of the acyl chain. **(F)** Chemical structure of a representative fatty acid. The blue dots represent the carbon position analyzed by ^1^H-NMR. Bar plots indicate mean ± SEM. Replicates of each cell line are shown as individual dots (n = 7). Statistical significance was assessed using one-way ANOVA. *P ≤ 0.05; **P ≤ 0.01; ***P ≤ 0.001; ****P ≤ 0.0001. MGLL, monoglyceride lipase; DGAT2, diacylglycerol O-acyltransferase 2; GPAT2, 1-acylglycerol-3-phosphate O-acyltransferase 2; GPAT4, glycerol-3-phosphate acyltransferase 4; AGPAT3, 1-acylglycerol-3-phosphate O-acyltransferase 3; AGPAT5, 1-acylglycerol-3-phosphate O-acyltransferase 5; MBOAT1, membrane-bound O-acyltransferase domain containing 1.

Intracellular glycerol is used as a building block for glycerophospholipids, which are the major lipids in cell membranes and can be incorporated into vesicles for either intracellular or extracellular transport. The glycerol moiety of triacylglycerols (and other acylglycerols) can be generated from three sources: glucose, glycerol, or other metabolites such as pyruvate, alanine, lactate, or TCA cycle intermediates via glyceroneogenesis (44, 45) (**Figure 4A**). Glycerol was identified, assigned, and quantified in several lipid classes. The ^1^H NMR peaks of the protons located at positions C1 and C3 of the glycerol backbone (CH_2_OR1–CHOR2–CH_2_OR3) of TAG generated two sets of peaks at 4.16 ppm and 4.45 ppm. The double doublet at 4.45 ppm was well resolved and isolated and thus integrated and used for quantification (**Figure 4B**). In addition, the glyceryl C3H_2_ group (CH_2_OR1–CHOR2-CH_2_–X) of glycerophospholipids was readily observed and used for quantification (**Figure 4C**). In both cases, R7shDEK cell lines harbored significantly increased levels compared with the R7 and R7sgRON cell lines. In contrast, when comparing the NMR peaks derived from free glycerol, both C1H_3_ and C3H_3_ and C2H_2_ resonances showed that the R7shDEK cells had decreased free glycerol levels compared with both the R7 and R7sgRON cell lines, and there was no difference between glycerol levels in R7 and R7sgRON cell lines (**Figure 4D**). These results suggest that DEK but not RON expression could lead to decreased levels of TAG and other glycerophospholipids and increased free glycerol levels.

Triacylglycerols (TAGs) are the major energy reservoir in animals. They contain three esters of glycerol with fatty acids (FA). The main source of free glycerol in cells is lipolysis, which occurs via the breakdown of triacylglycerols into glycerol and free fatty acids (FFAs). Glycerol can also be produced directly from glycerol-3-phosphate via glycolysis (46) (**Figure 4A**). FAs, specifically the fatty acyl chains as components of complex lipids, are essential for maintaining cell membrane architecture. In fact, *de novo* FA synthesis (lipogenesis) has been recognized as a hallmark of malignancy. In breast cancer, FA synthesis was reported to be heightened via the upregulation of several lipogenic enzymes, such as FASN and ACLY (47–49).

The main substrate for the biosynthesis of FA is acetyl-CoA, which is first carboxylated by acetyl-CoA carboxylase to form malonyl-CoA. The condensation of seven molecules of malonyl-CoA and one molecule of acetyl-CoA ultimately generates palmitate, a 16-carbon saturated FA. Palmitoyl-CoA is the main product of lipogenesis and can later be elongated and desaturated to produce other FA species. In cancer cells, as a consequence of increased *de novo* lipogenesis, saturated and monounsaturated FA are the dominant species of FA (12). This provides an advantage for cancer progression and metastasis, since these forms of FA are more stable, less susceptible to peroxidation, and ultimately more resistant to cellular damage (50). Hence, the composition of these FAs is also decisive for the survival of tumor cells. The decreased level of unsaturation in membranes may affect their properties and those of integral membrane proteins.

Quantification of the olefinic (C=CH), as well as the bis-allylic (–CH_2_) group, which is specific for linoleic acid, revealed that R7shDEK cells harbor significantly increased levels of unsaturated FAs (**Figures 4E, F**) compared with the R7 and R7sgRON cell lines. This suggests that DEK expression decreases unsaturated FAs, thus decreasing membrane fluidity. In addition, the analysis of the α-methylene FA group showed that total FA levels are increased in R7shDEK compared with R7 and R7sgRON. GPATs (glycerol-3-phosphate acyltransferases) catalyze the first step of the *de novo* biosynthesis of TAG and glycerophospholipids, whereas the AGPATs (acylglycerolphosphate acyltransferase) and MBOAT1 (membrane-bound O-acyltransferase domain containing 1) catalyze the second step (51). These enzymes have a central role as modulators of lipid homeostasis and storage (52, 53). Specifically, downregulation of GPAT2 has been associated with malignancy, proliferation, and survival in breast cancer cell lines (54). Inhibition of MGLL (monoacylglycerol lipase), which is an enzyme involved in the generation of fatty acids and glycerophospholipids from monoacylglycerol, was associated with decreased infiltration into the blood–brain barrier in triple-negative breast cancer in mouse models (55). This contrasts with our findings below that decreased MGLL mRNA levels are associated with worse prognosis in breast cancer. However, it is important to note that mRNA levels do not necessarily reflect protein levels and/or enzyme activity (56).

### 3.5 A gene signature of lipid metabolic enzymes predicts breast, ovarian, and lung cancer outcome

**Figure 5:**
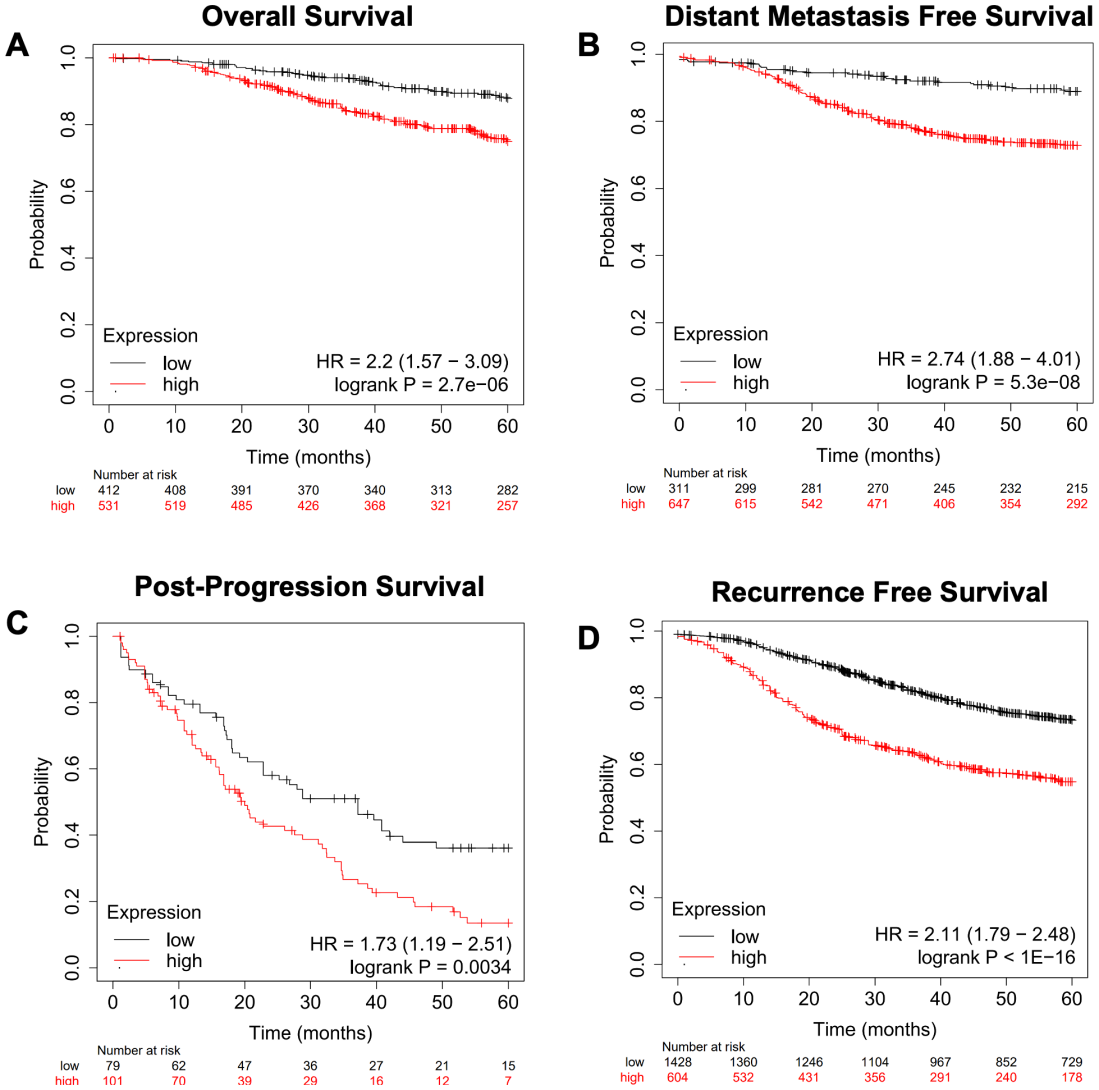
A gene expression signature for lipid metabolism based on KMplot.com GEO-derived breast cancer dataset. Gene Expression Omnibus-derived KMplot breast cancer datasets (23) were used to query. **(A)** Overall survival (OS), **(B)** distant metastasis-free survival (DMFS), **(C)** post-progression survival (PPS), and **(D)** recurrence-free survival (RFS) of breast cancer patients stratified by expression of the lipid gene signature (**Table 1**).

We next focused on genes involved in the metabolism of various lipid classes (fatty acids, cholesterol, steroids, sphingolipids, phospholipids, triglycerides, glycolipids, and cardiolipins) and determined where the expression of genes encoding metabolic enzymes in these pathways was associated with worse outcomes in breast cancer outside of the RON/DEK context. We focused on relapse-free survival (RFS) since breast cancer outcomes worsen substantially upon relapse. Using the KMplot webtool, we investigated overall survival (OS, **Figure 5A**), distant metastasis-free survival (DMFS, **Figure 5B**), post-progression survival (PPS, **Figure 5C**), and recurrence-free survival (RFS, **Figure 5D**) from the Gene Expression Omnibus (GEO)-derived dataset and identified a gene signature of 47 genes associated with significantly decreased patient survival regardless of breast cancer subtype. **Table 1** shows the panel of genes, the respective lipid group associated with each gene, and up- or downregulation in patients with worse outcomes. Genes that are predictive of poor patient outcomes when upregulated include the following: enzymes involved in the hydrolyzation of acyl-CoA molecules, such as ACOT7 and ACOT13 (57, 58); DHCR7, an enzyme involved in the final steps of cholesterol biosynthesis (59); and ARSK, an enzyme involved in the modification of steroids, glycolipids, and carbohydrates (60). Genes that are predictive of good patient outcomes when upregulated include ELOVL5, an enzyme involved in the elongation of fatty acids (61); BDH2, an enzyme involved in fatty acid beta oxidation (62); ETNK1, an enzyme required for the first step of phosphatidylethanolamine synthesis (63); and MGLL, an enzyme involved in the production of fatty acids and glycerol from acylglycerides (64).

**Table 1:** Lipid metabolism genes with prognostic capacity.

Lipid class	Gene	Upregulated (Up) or downregulated (Down)	Log-rank p-value			
			RFS	OS	DMFS	PPS
Fatty acids	ACOT7	Up	9e−05	0.055	0.031	0.0014
	ACOT13	Up	1.7e−08	0.071	0.00024	0.045
	ME1	Up	2.9e−15	0.001	4.3e−08	0.02
	ANGPTL4	Up	5.7e−11	1.8e−06	4.9e−07	0.083
	OLAH	Down	0.018	0.12	0.31	0.09
	ACACB	Down	<1e−16	0.0022	6e−10	0.12
	IVD	Down	3.1e−16	0.00043	0.00057	0.037
	FASN	Down	3.5e−06	0.018	5.7e−05	0.022
	ACSL6	Down	7.2e−06	0.00033	0.0027	0.12
	ELOVL5	Down	<1e−16	6.3e−07	<1e−16	0.00013
	CPT1C	Down	3.1e−05	0.092	0.043	0.031
	CPT1B	Down	1.4e−15	0.012	0.14	0.016
	ACADSB	Down	<1e−16	1.8e−10	1.3e−09	0.023
	ACAA2	Down	2.8e−05	0.3	0.037	0.038
	ACOX1	Down	0.0074	0.094	0.13	0.14
	PTGR1	Down	1.9e−05	0.0024	0.007	0.0019
	FAAH	Down	<1e−16	0.0089	2.1e−05	0.18
	ACSF2	Down	<1e−16	2.8e−05	9.1e−11	0.00033
	BDH2	Down	6.9e−11	0.0018	6.5e−05	0.042
	ACOT11	Down	9e−05	0.055	0.031	0.0014
	MBOAT1	Down	2.3e−15	5e−07	1.5e−05	0.085
Cholesterol	DHCR7	Up	3.3e−15	8.5e−09	9.7e−10	0.0017
	MVK	Down	3e−14	0.062	0.0011	0.11
	STARD3	Down	6.8e−06	1e−04	0.0023	0.00088
	OSBPL5	Down	9.4e−12	0.01	0.062	0.36
Steroid	SRD5A1	Up	9.5e−14	0.0015	2.4e−10	0.0019
	HSD17B2	Up	5.3e−05	2.7e−05	3.9e−05	0.0039
	NCOA1	Down	3e−10	0.00078	1.3e−06	0.16
Sphingolipids	UGT8	Up	3.4e−05	0.0061	0.021	0.019
Phospholipids	LCLAT1	Up	1.8e−05	0.079	0.035	0.26
	PLAAT1	Up	<1e−16	3.8e−05	2.2e−12	0.0057
	AGPAT3	Down	7e−12	0.0046	0.0017	0.29
	AGPAT5	Down	1.1e−06	0.0012	0.051	0.028
	GPAT4	Down	2.8e−12	0.24	0.28	0.31
	INPP4B	Down	3e−12	0.0034	8.8e−10	0.025
	ETNK1	Down	1.4e−05	0.00041	0.022	0.36
	PLAAT5	Down	1.1e−10	0.0064	0.00037	0.068
	PITPNM2	Down	6e−09	0.0011	0.052	0.0028
	SLC44A4	Down	<1e−16	0.00052	1.2e−09	0.14
Triglycerides	DGAT2	Up	0.0021	0.00033	0.0071	0.016
	APOA5	Down	7.8e−08	0.026	0.074	0.021
	GPAT2	Down	0.0025	0.34	0.023	0.037
	MGLL	Down	0.003	0.0022	0.21	0.24
Glycolipids	ARSK	Up	5.7e−14	0.036	0.032	0.11
	NEU4	Up	4.4e−06	0.014	0.041	0.16
Cardiolipins	PLD6	Down	9.9e−08	0.13	0.019	0.014

Genes are displayed with a notation of upregulated (Up) or downregulated (Down) in breast cancer in association with worse outcomes. The log-rank p-value for Kaplan–Meier survival curves obtained from the KMplot webtool (23) are included for relapse-free survival (RFS), overall survival (OS), distant metastasis-free survival (DMFS), and post-progression survival (PPS).

**Figure 6:**
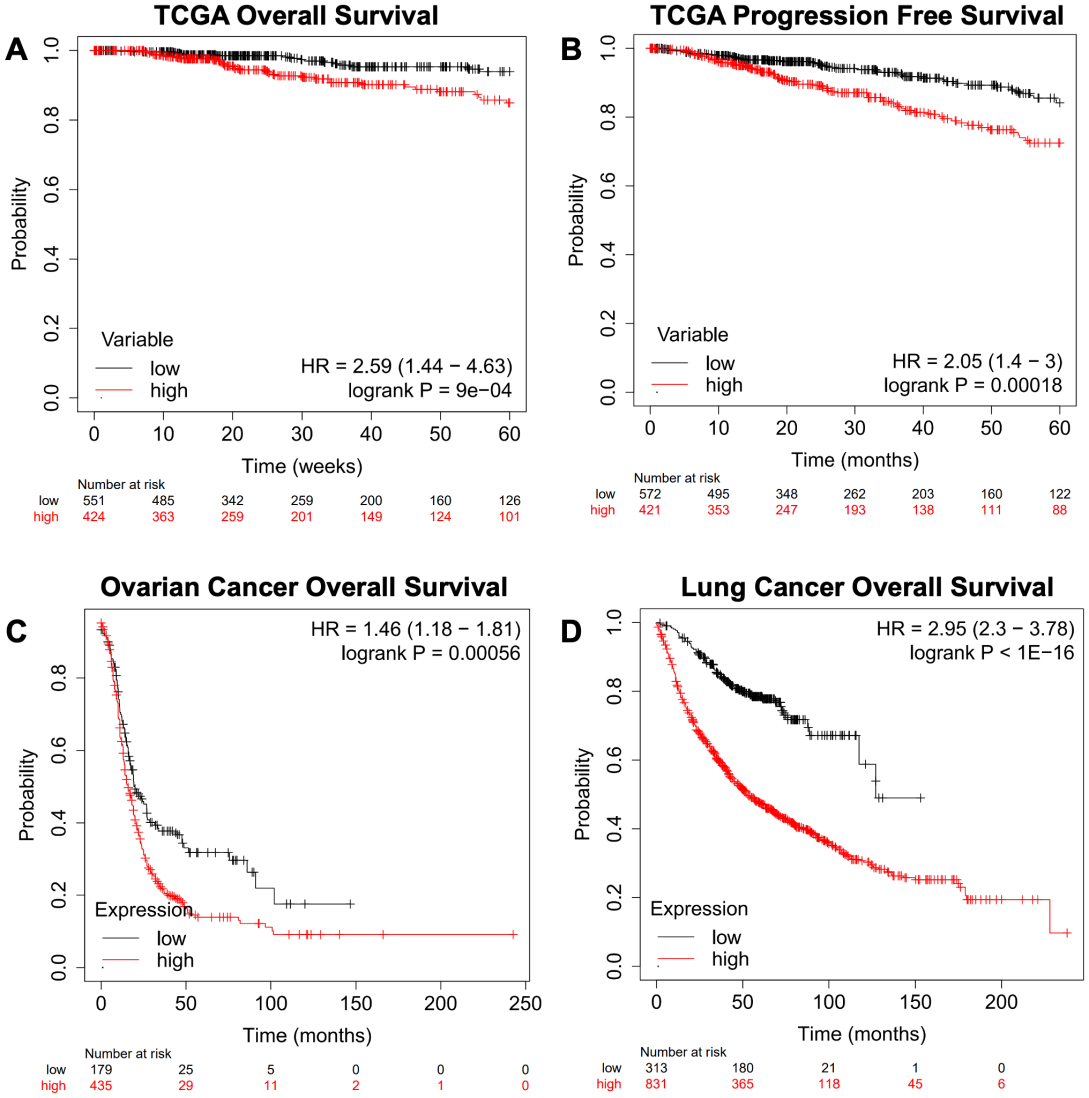
Confirmation of a gene expression signature via a TCGA-based breast cancer dataset and KMplot.com ovarian and lung cancer. The gene signature developed on the KMplot cancer datasets (**Table 1**) was tested against a breast cancer dataset from The Cancer Genome Atlas (TCGA) Pan-Cancer datasets to examine **(A)** overall survival (OS) and **(B)** progression-free survival (PFS). Additionally, Gene Expression Omnibus-derived KMplot ovarian cancer **(C)** and lung cancer **(D)** datasets (26, 27) were used to query the effectiveness of the gene signature in cancers other than breast cancer via overall survival (OS).

We next queried the gene signature using an independent Breast Cancer dataset derived from TCGA Pan-Cancer Atlas using overall survival (OS, **Figure 6A**) and progression-free survival (PFS, **Figure 6B**), as well as Ovarian Cancer (**Figure 6C**) and Lung Cancer datasets (**Figure 6D**) (26). This analysis recapitulated the results obtained from the dataset used in the KMplot webtool.

**Figure 7:**
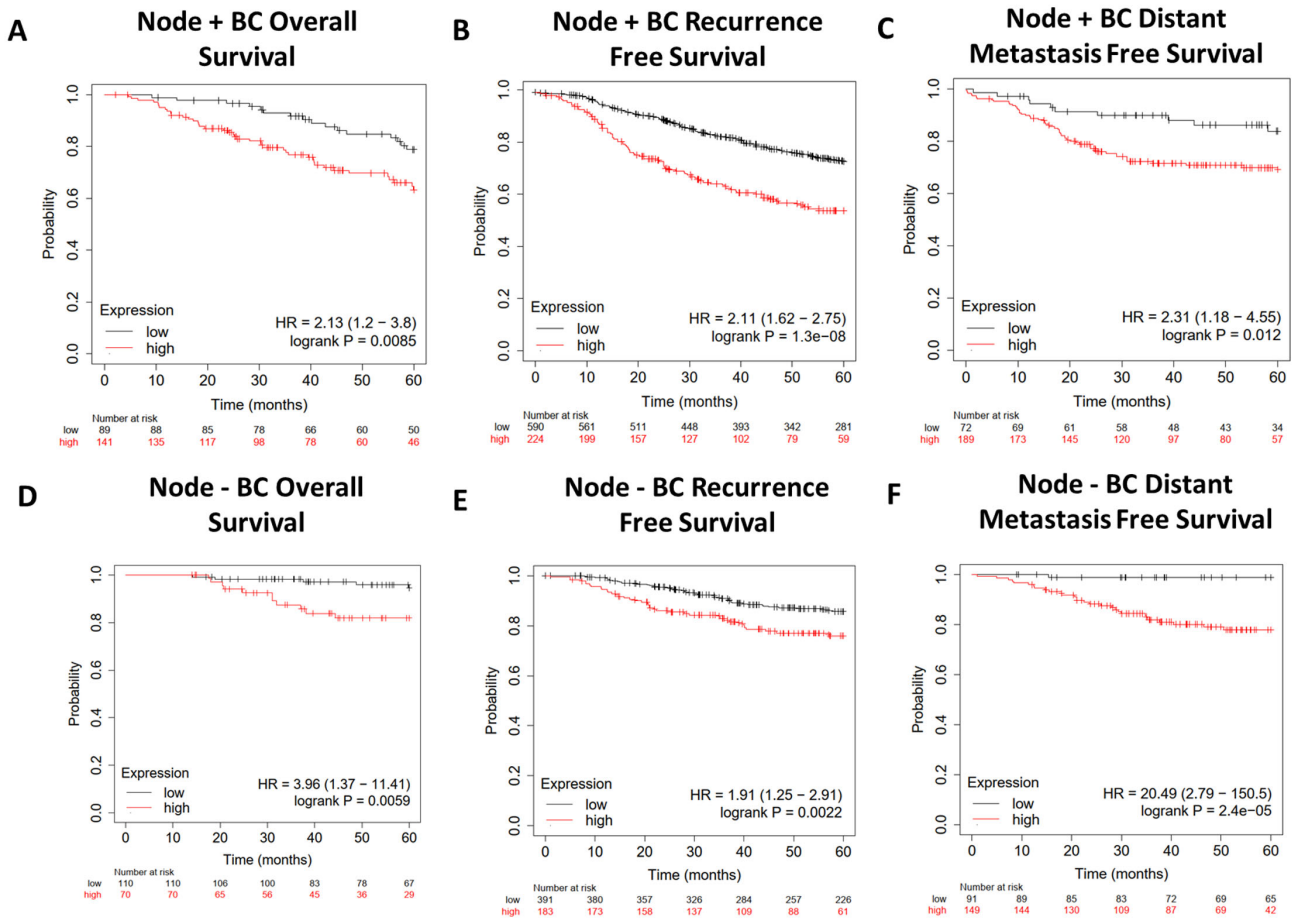
Survival analysis of Node+ and Node− breast cancer patients from GEO-derived datasets on KMplot.com. Gene Expression Omnibus-derived KMplot breast cancer datasets (23) were used to query **(A, D)** overall survival (OS), **(B, E)** recurrence-free survival (RFS), and **(C, F)** distant metastasis-free survival (DMFS) of breast cancer patients stratified by expression of the lipid gene signature (**Table 1**).

We further characterized the lipid gene signature in breast cancer by narrowing our focus to either Node+ or Node− patients using data from KMplot.com (**Figure 7**). The gene signature predicted worse overall survival (**Figures 7A, D**), recurrence-free survival (**Figures 7B, E**), and distant metastasis-free survival (**Figures 7C, F**) regardless of nodal status, with a hazard ratio of 20.49 for distant free metastasis in Node− patients (**Figure 7F**). Importantly, this implies that in patients with early-stage (Node−) breast cancer, this gene signature is highly predictive of progression to metastatic disease.

**Figure 8:**
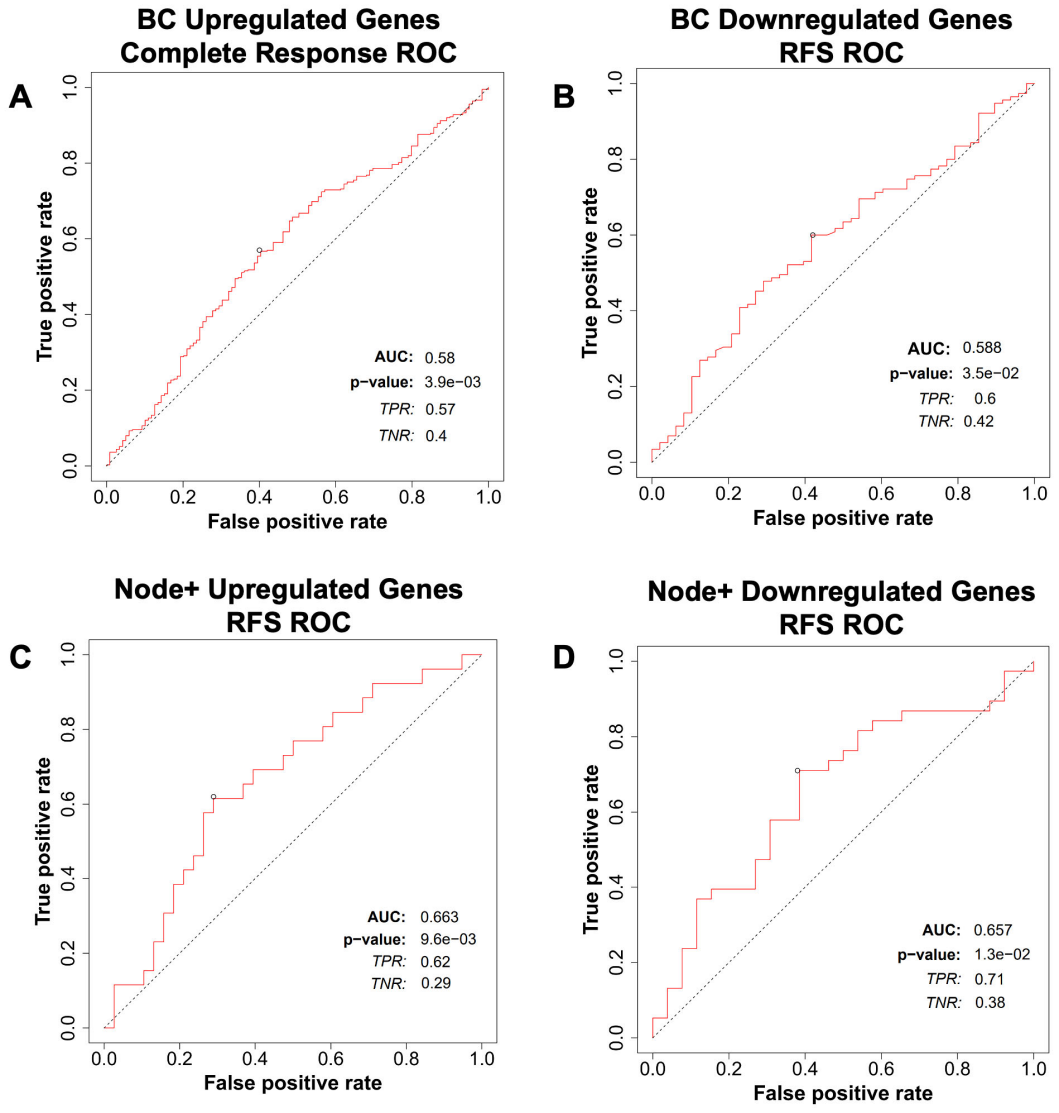
Breast cancer patient response to therapy compared with the gene expression signature from ROCplot.com. **(A)** Receiver-operator characteristic (ROC) analysis of breast cancer patient complete response to chemotherapy stratified by expression of genes in the lipid gene signature (**Table 1**) found to be upregulated in recurrent breast cancer. **(B)** Receiver-operator characteristic (ROC) analysis of breast cancer patient recurrence-free survival (RFS) to chemotherapy stratified by expression of genes in the gene signature found to be downregulated in recurrent breast cancer. Receiver-operator characteristic (ROC) analysis of node-positive breast cancer patient RFS to therapy stratified by expression of genes in the gene signature found to be **(C)** upregulated and **(D)** downregulated in recurrent breast cancer.

We then tested the predictive potential of the above lipid metabolism signature for breast cancer, ovarian cancer, colorectal cancer, and glioblastoma response to therapy using the ROCplot webtool. The genes were separated into two sets. One set included genes that were overexpressed in cancers that had recurred, whereas the other set included genes that were underexpressed in cancers that had recurred. Genes that were overexpressed were predictive of a complete response to chemotherapy in breast cancer (**Figure 8A**), whereas genes that were underexpressed were predictive of relapse-free survival in breast cancer (**Figure 8B**). Both sets of genes were predictive of relapse-free survival in Node+ breast cancer (**Figures 8C, D**).

**Figure 9:**
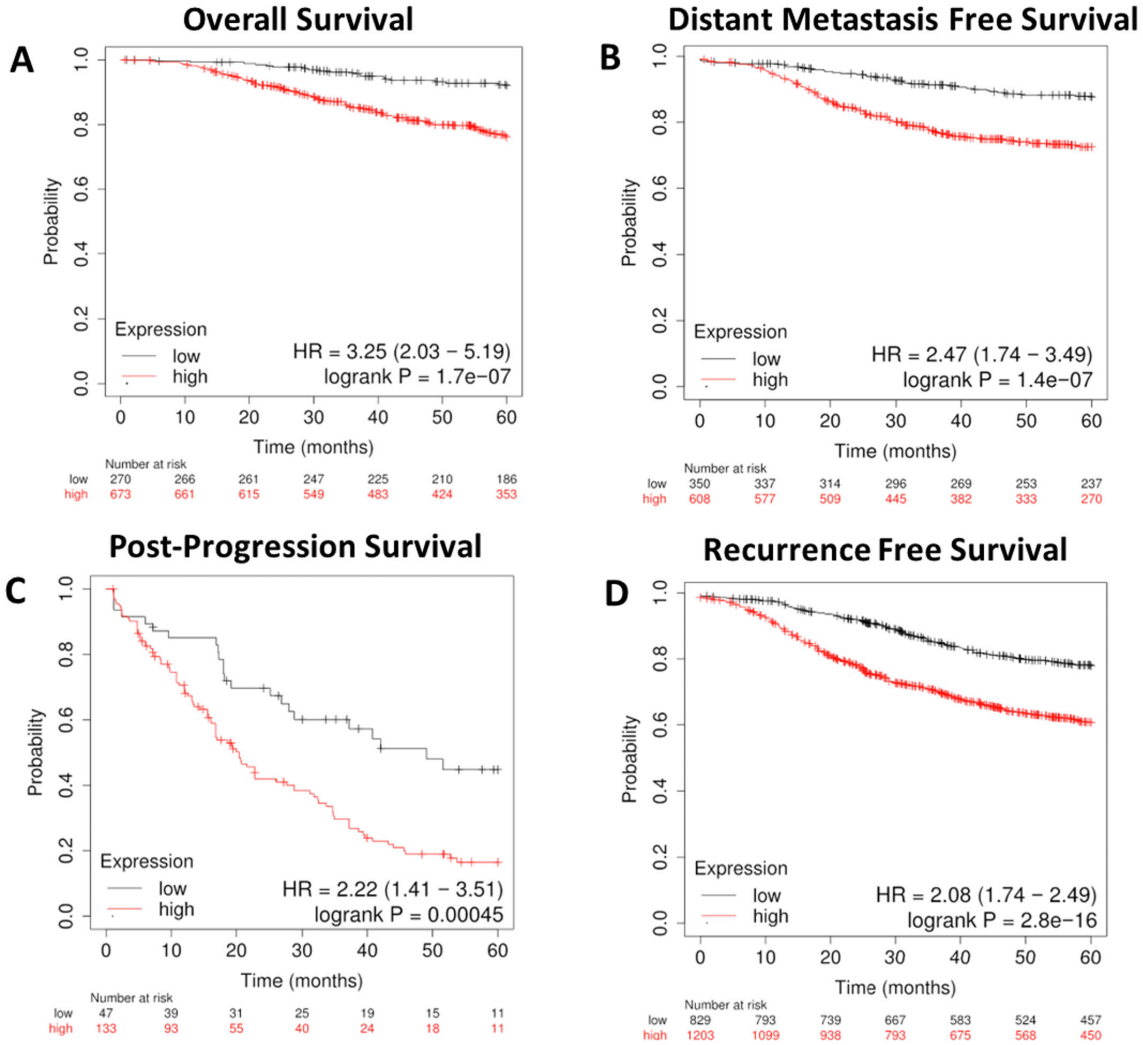
A refined gene expression signature for lipid metabolisms based on KMplot.com GEO-derived breast cancer dataset. Gene Expression Omnibus-derived KMplot breast cancer datasets (23) were used to query **(A)** overall survival (OS), **(B)** distant metastasis-free survival (DMFS), **(C)** post-progression survival (PPS), and **(D)** recurrence-free survival (RFS) of breast cancer patients stratified by expression of the four gene lipid gene signature (**Table 2**).

To further refine the lipid gene signature to its necessary components, we determined the minimal number of lipid metabolism related genes predictive of breast cancer outcomes and arrived at four genes based on their RNA-seq expression in R7 and T47D cell lines compared with their isogenic shRON counterparts (65): DHCR7, BDH2, ELOVL5, and ARSK. Compared with the above signature of 47 genes, these four genes were equally sufficient to predict poor overall survival, distant metastasis-free survival, post-progression survival, and recurrence-free survival at 60 months (**Figures 9A–D**). In addition, ROC analysis was performed using these four genes on the ROCplot webtool showing a prediction of complete response to chemotherapy similar to that of the 47 gene signature (**Supplementary Figure S2**).

**Table 2:** Four lipid metabolism genes with prognostic capacity.

Gene	Upregulated (Up) or downregulated (Down)
ELOVL5	Down
BDH2	Down
DHCR7	Up
ARSK	Up

Genes are displayed with a notation of upregulated (Up) or downregulated (Down) in breast cancer in association with worse outcomes.

## 4 Discussion

RON and DEK are implicated in breast cancer metastasis and recurrence, and since these same phenotypes have been linked to lipid dysregulation, the gene signature discovered here might have diagnostic and prognostic potential for patients with breast and other cancer types. In the present study, ^1^H-NMR spectroscopy was employed as an exploratory tool to identify dysregulated lipid families and characterize the lipid metabolic profile of murine breast cancer cells with respect to loss of RON or DEK expression. Specifically, we obtained global information on glycerophospholipid head groups and backbones, acyl chain length, and degree of unsaturation of fatty acids, as well as cholesterol and sphingolipid content. Although NMR-based methods are generally less sensitive than MS and can be limited by overlapping signals, here we describe a simple and efficient NMR-based lipidomic method to provide a non-destructive comprehensive lipid profile in a single experiment with minimal sample processing and without multiple internal standards.

A wealth of evidence has shown that lipid metabolism is important in metastasis and recurrence of breast cancer. For instance, cholesterol biosynthesis inhibition using statins lowered the risk of recurrence (66). We have further shown that RON signaling increases cholesterol levels and that RON-stimulated metastasis and recurrence can be suppressed by statins. In addition, fatty acid synthesis promotes DNA damage and ROS production in residual breast cancer cells (67). Although the exact roles that DNA damage and ROS play in cancer are unclear, the data presented in the gene signature here imply that fatty acid synthesis may suppress cancer progression, perhaps due to modulation of DNA damage and ROS levels. Independent of RON, DEK appears to lower total fatty acid levels, as a possible contributor to the pro-tumor capabilities of DEK in breast cancer. Although clinical data are lacking, trends in the published cellular literature indicate that sphingolipids and phospholipids also play key roles in breast cancer recurrence and metastasis (68–70). RON, independent of DEK, seems to increase sphingomyelin levels. The literature combined with the lipid data presented in this report supports possible roles for RON and DEK in the reprogramming of breast cancer cells toward a cancer stem cell phenotype. Specifically, RON appears to promote cholesterol and sphingomyelin levels whereas DEK suppresses cholesterol and total fatty acid levels. However, further investigations are necessary to establish the functional consequences of the observed lipid profile.

We have previously reported studies of the polar fractions from the above cell lines (18). Integrating the polar and non-polar data increases our understanding of the impact of RON and DEK on pathways that feed into lipid metabolism. For instance, we previously found that pyruvate levels were suppressed by RON but stimulated by DEK. Pyruvate can be metabolized into glycerol, which also shows a similar pattern as pyruvate with suppression by RON, and stimulation by DEK in the absence of glycerol changes. This indicates that RON and DEK may independently regulate the flux of pyruvate into different pathways. In addition, pyruvate can be converted into acetyl-CoA and used for fatty acid and cholesterol synthesis. Other contributors to acetyl-CoA levels that we have previously measured include isoleucine, which was found to be increased by both DEK and RON; glutamate, which was not affected by RON or DEK knockdown; citrate, which was found to be suppressed by both RON and DEK; and acetate, which was found to be increased by both RON and DEK. However, in the absence of tracer experiments, it is difficult to determine the relative contributions of these different pathways to lipid synthesis.

For phospholipids, we found in our previous manuscript that phosphatidylcholine levels were stimulated by RON and DEK, whereas glycerophosphatidylcholine was suppressed by RON and stimulated by DEK. The observed dysregulation of specific phospholipids in the absence of RON occurred without any change in total phospholipid levels, and the lack of a change in the glycerol signature. In addition, we observed alterations in sphingolipid levels, the degree of unsaturation, and cholesterol level, which is importantly involved in membrane lipid raft function. This suggests that RON and DEK may control pathways that influence the physical properties of membranes including fluidity and signaling, important possible contributors to metastasis and recurrence in breast cancer.

There is evidence that implicates ELOVL5 (61), DHCR7 (71), ACOT7 (72), ME1 (73), ACACB (74), FASN (75), ACSL6 (76), CPT1B (77), FAAH (78), ANGPTL4 (79), STARD3 (80), SRD5A1 (81), HSD171B (82), NCOA1 (83), UGT8 (40), INPP4B (84), DGAT2 (85), MBOAT1 (86), PTGR1 (87), and PLD6 (88) in breast cancer prognosis. However, these studies focused on changes in growth, proliferation, and prognosis and did not report on changes in metabolites. Several genes included in the predictive lipid-related gene signature have not yet been studied in the context of breast cancer, which include ACAA2, ACSF2, AGPAT3, AGPAT6, APOA5, ARSK, BDH2, CBR4, ACOT13, ACOT11, MVK, OLAH, ACADSB, ACOX1, IVD, LCLAT1, MGLL, SLC44A4, PLAAT1, PLAAT5, and PITPNM2. This indicates a need for additional studies of the functional roles of these genes as potential drivers of breast cancer metastasis and recurrence. For example, the hazard ratio for the ACADSB RFS curve alone was 0.54, which indicates that patients with high ACADSB levels in their tumors are half as likely to have recurrence compared with patients with low ACADSB, thus supporting a potential high value of this gene as a predictive biomarker and driver or survival.

To define the significance of the gene signature, we focused on node +ve breast cancers, which have already spread to lymph nodes and carry a high likelihood of recurrence. Focusing on the group of 47 genes (**Table 1**) that were indicative of improved response to treatment in node +ve patients, we also identified patients at a higher risk of recurrence and non-response to treatment. We were able to narrow this gene signature down to a combination of only four genes which fully retain the predictive power of the previous signature. At least some of these genes might be functionally involved in cancer phenotypes that portend poor outcome, and in this case, their therapeutic modulation may result in new avenues to suppress breast cancer recurrence and/or treatment resistance. Lipid profiles identified in sgRON and shDEK murine cell lines are the foundation for future research in animal models of breast cancer recurrence and metastasis. Data validation in human models is now critical to advance lipid related biomarkers and targets toward clinical application.

## Data Availability

The original contributions presented in the study are included in the article/**Supplementary Material**, while the raw datasets are available in a publicly accessible repository. This data can be found at the NIH Common Fund's National Metabolomics Data Repository. Project ID PR002009.

## Supplementary material

Supplementary Figure 1 Multivariate statistical analysis by principal component analysis (PCA) distinguishes cell lines based on their lipid profile. **(A)** PCA scatter plot shows distinct clustering of the three cell lines (R7, R7sgRON and R7shDEK) according to their lipid composition, indicating a clear difference in their lipidomes. **(B)** Loading plot where each variable is represented as a vector, and the length of the vector indicates the influence of that variable in the model. Variables with longer vectors are more influential, whereas variables with shorter vectors have less impact on the model. **(C–E)** PCA scatterplot for individual cell lines: **(C)** R7 (n=7), **(D)** R7sgRON (n=7), **(E)** R7shDEK (n=7). Prior to PCA, the data were scaled to unite variance to ensure equal contribution from each variable. This distinct clustering pattern suggests that the cell lines exhibit unique lipid signature.

Supplementary Figure 2 Breast Cancer Patient Response to therapy compared with the four gene expression signature from ROCplot.com. Receiver-operator characteristic (ROC) analysis of breast cancer patient complete response to chemotherapy stratified by expression of genes in the four gene signature (**Table 2**).
