# Supplementary figures and images for: Lipid profiling of RON and DEK-dependent signaling in breast cancer guides discovery of gene networks predictive of poor outcomes
Source: Front Oncol. 2024 Sep 16;14:1382986. doi: 10.3389/fonc.2024.1382986 (PMC11440356; doi:10.3389/fonc.2024.1382986)

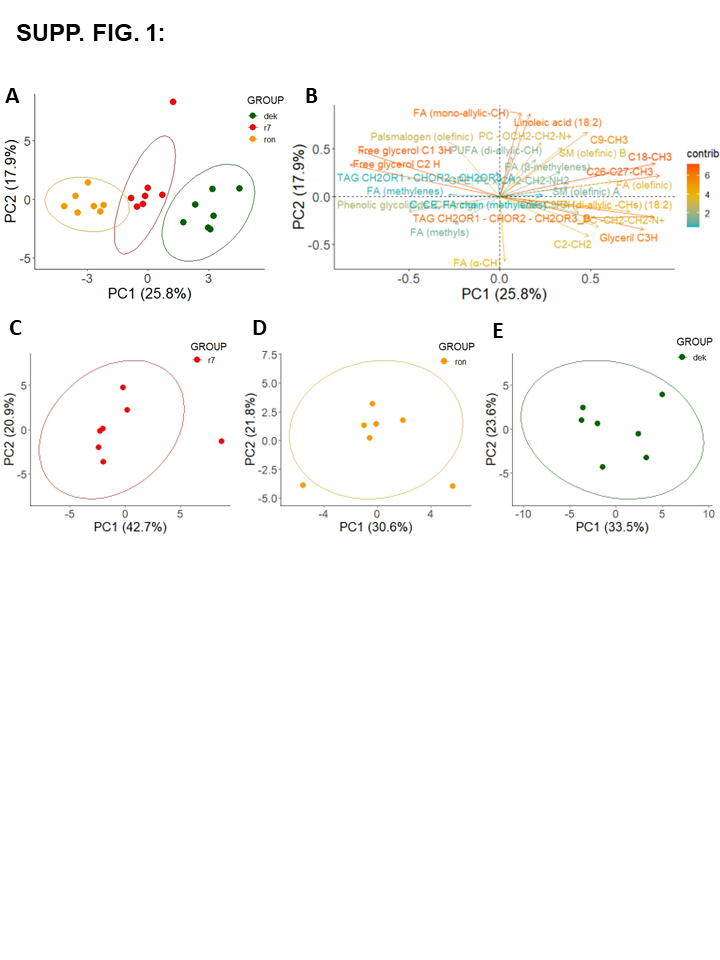

Supplement: Supplementary Figure 1 — Multivariate statistical analysis by principal component analysis (PCA) distinguishes cell lines based on their lipid profile. (A) PCA scatter plot shows distinct clustering of the three cell lines (R7, R7sgRON and R7shDEK) according to their lipid composition, indicating a clear difference in their lipidomes. (B) Loading plot where each variable is represented as a vector, and the length of the vector indicates the influence of that variable in the model. Variables with longer vectors are more influential, whereas variables with shorter vectors have less impact on the model. (C–E) PCA scatterplot for individual cell lines: (C) R7 (n=7), (D) R7sgRON (n=7), (E) R7shDEK (n=7). Prior to PCA, the data were scaled to unite variance to ensure equal contribution from each variable. This distinct clustering pattern suggests that the cell lines exhibit unique lipid signature. [file Image1.tif]

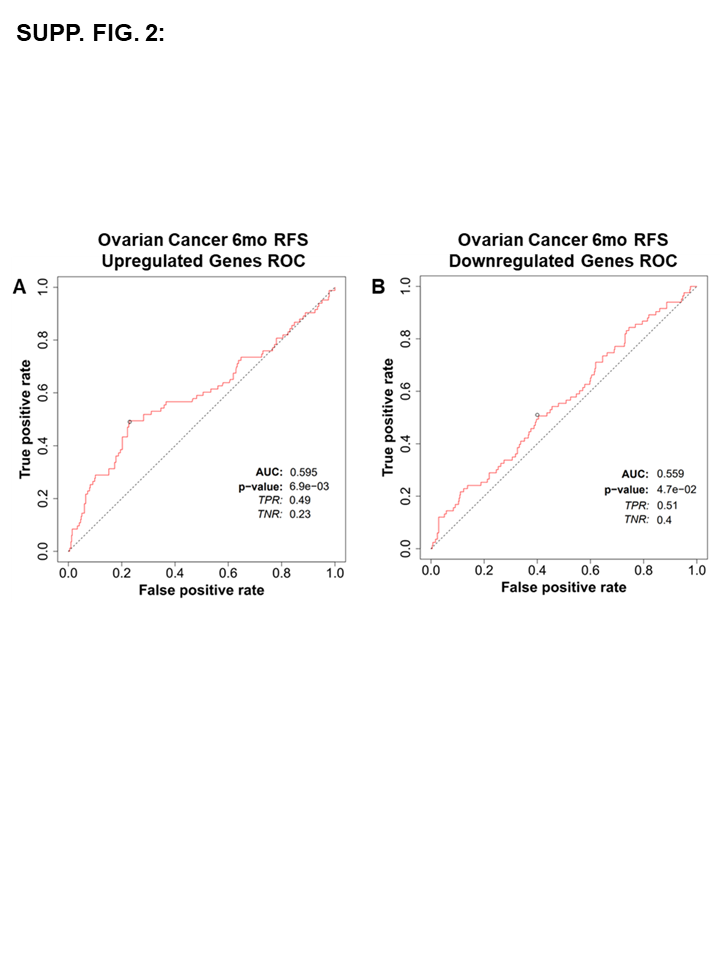

Supplement: Supplementary Figure 2 — Breast Cancer Patient Response to therapy compared with the four gene expression signature from ROCplot.com. Receiver-operator characteristic (ROC) analysis of breast cancer patient complete response to chemotherapy stratified by expression of genes in the four gene signature ( Table 2 ). [file Image2.tif]
